# Nanostructural and Transcriptomic Analyses of Human Saliva Derived Exosomes

**DOI:** 10.1371/journal.pone.0008577

**Published:** 2010-01-05

**Authors:** Viswanathan Palanisamy, Shivani Sharma, Amit Deshpande, Hui Zhou, James Gimzewski, David T. Wong

**Affiliations:** 1 Department of Craniofacial Biology, College of Dental Medicine, Medical University of South Carolina, Charleston, South Carolina, United States of America; 2 Department of Chemistry and Biochemistry, University of California Los Angeles, Los Angeles, California, United States of America; 3 California NanoSystems Institute, University of California Los Angeles, Los Angeles, California, United States of America; 4 School of Dentistry and Dental Research Institute, University of California Los Angeles, Los Angeles, California, United States of America; 5 International Center for Materials Nanoarchitectonics Satellite (MANA), National Institute for Materials Science (NIMS), Tsukuba, Japan; 6 Molecular Biology Institute, University of California Los Angeles, Los Angeles, California, United States of America; 7 Division of Head and Neck Surgery/Otolaryngology, School of Medicine, University of California Los Angeles, Los Angeles, California, United States of America; 8 Henry Samuel School of Engineering and Applied Science, University of California Los Angeles, Los Angeles, California, United States of America; 9 Jonsson Comprehensive Cancer Center, University of California Los Angeles, Los Angeles, California, United States of America; New Mexico State University, United States of America

## Abstract

**Background:**

Exosomes, derived from endocytic membrane vesicles are thought to participate in cell-cell communication and protein and RNA delivery. They are ubiquitous in most body fluids (breast milk, saliva, blood, urine, malignant ascites, amniotic, bronchoalveolar lavage, and synovial fluids). In particular, exosomes secreted in human saliva contain proteins and nucleic acids that could be exploited for diagnostic purposes. To investigate this potential use, we isolated exosomes from human saliva and characterized their structural and transcriptome contents.

**Methodology:**

Exosomes were purified by differential ultracentrifugation and identified by immunoelectron microscopy (EM), flow cytometry, and Western blot with CD63 and Alix antibodies. We then described the morphology, shape, size distribution, and density using atomic force microscopy (AFM). Microarray analysis revealed that 509 mRNA core transcripts are relatively stable and present in the exosomes. Exosomal mRNA stability was determined by detergent lysis with RNase A treatment. *In vitro*, fluorescently labeled saliva exosomes could communicate with human keratinocytes, transferring their genetic information to human oral keratinocytes to alter gene expression at a new location.

**Conclusion:**

Our findings are consistent with the hypothesis that exosomes shuttle RNA between cells and that the RNAs present in the exosomes may be a possible resource for disease diagnostics.

## Introduction

Exosomes-small nanovesicles released from various cells have increasingly studied for their potential use in therapeutics and diagnostics [1], [2]. Exosomes are derived from endosomal membrane compartments after fusion with the plasma membrane and are released from activated cell surfaces [3], [4], [5]. These exosomes, or microvesicular bodies (MVBs), are produced by different cell types, including dendritic cells [6], macrophages [7], and lymphocytes [8], as well as salivary gland epithelial cells [9] and tumor cells [10]. Exosomes have been found in physiological fluids such as saliva [11], [12], plasma [2], urine [13], amniotic fluid [14], malignant ascites [15], bronchoalveolar lavage fluid [16], synovial fluids [17], and breast milk [18]. Although exosomes have been identified in human saliva, their biochemical and biophysical characteristics are largely unknown. Due to their small size, morphological analysis of purity and exosome characterization has solely been limited to electron microscopy (EM). Here, we employed atomic force microscopy (AFM) to more thoroughly characterize native exosomes without the need for fixation, staining, or labeling of these particles. AFM has previously been used to yield nanometer-scale topographical images of biological molecules [19]. Using AFM, we showed a 3D structure of exosomes from human saliva. To the best of our knowledge, such a structure has not been reported previously for any exosome.

Messenger RNA profiling of saliva from both healthy individuals and those with oral cancer has led to the nucleic acid characterization of human saliva, and RNA enrichment in saliva holds the promise of salivary biomarkers as future tools [20], [21], [22], [23]. Several studies suggest that exosomes may stimulate target cells and transfer surface receptors and genetic information [24], [25], [26]. In fact, exosomes were shown to transfer surface molecules, tumor cell mRNA, and infective agents such as HIV or prions [27], [28]. In addition, Valadi and colleagues [29] demonstrated that secretory exosomes released from mast cells *in vitro* contain not only proteins but also a population of mRNA and miRNA. Recently, exosomes derived from glioblastoma tumor cells and blood from cancer patients were reported to promote tumor growth and to contain mRNA and proteins useful for diagnostic biomarkers [30]. These exosomes are attractive vesicles for intercellular mRNA transfer because they likely provide a protected environment to ensure stability in the presence of extracellular RNases. Interestingly, Deregibus and colleagues isolated microvesicles containing mRNA from endothelial precursor cells and verified their transfer using GFP-tagged mRNA [24]. Additionally, Smalheiser and colleagues showed that exosomes transfer synaptic proteins such as CAM kinase II alpha and synaptic mRNA to the presynaptic terminal, where these factors contribute to synaptic plasticity [31]. Although exosome mRNA has been implicated in cell-cell signaling, the characteristic features and transfer abilities of these vesicles are largely unknown in body fluid such as saliva.

An intriguing aspect of the present work is the mechanism of RNA targeting to exosomes. MVBs, the organelles from which exosomes are derived, generated from the fusion of early endosomes and have a well established role in the degradation of proteins internalized from the cell surface via fusion with lysosomes [28]. In addition to fusion with lysosomes, MVBs also undergo exocytotic fusion with the plasma membrane and release their “intraluminal vesicles,” which refers to the exosomes that are contained within the MVBs [32]. Possibly, RNAs may initially be internalized in the cytoplasm via early endosomes and subsequently incorporated into MVBs before being secreted through the fusion of MVBs with the plasma membrane. The accumulation of RNA in exosomes is a concept that has not been investigated thoroughly. Thus, we hypothesize that saliva RNA and proteins are secreted via the process of exosome formation. More specifically, delivery of the exosomes to the oral cavity occurs by fusion of the MVB outer membrane with the plasma membrane of oral epithelial cells. Thus, saliva exosomes should have the characteristic features of internal vesicles of MVBs [26], and the vesicles should be small (<100 nm) and relatively uniform in size, similar to other exosomes [33] secreted by other cells and tissues. In addition, saliva exosomes should contain proteins like CD63 and Alix, which is typical of MVBs and other exosomes [29], along with genetic information. Finally, saliva exosomes should be capable of communicating with neighboring cells such as human oral keratinocytes and altering gene expression at the new location.

## Results

### Evidence of Exosomes in Human Saliva

**Figure 1:**
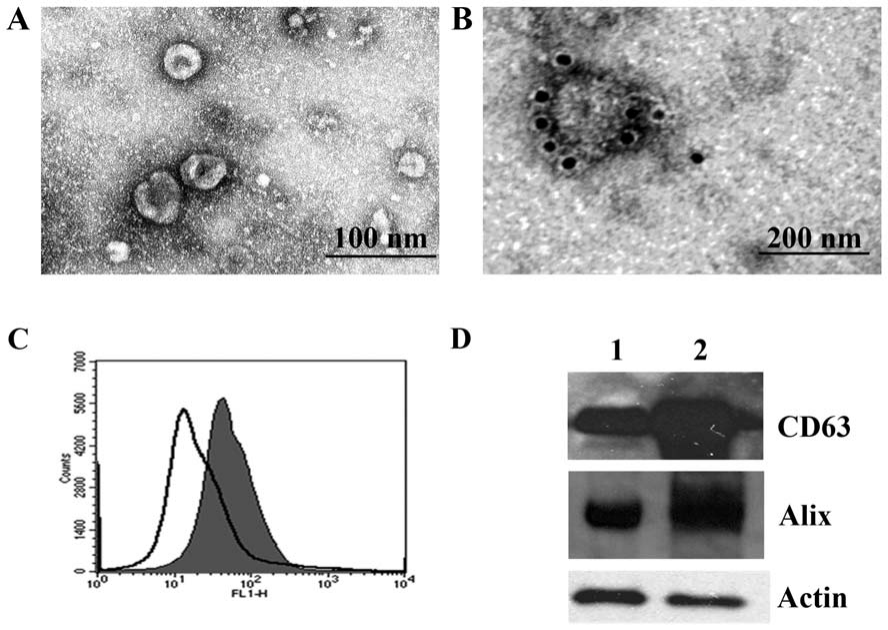
EM image of human saliva showing round-shaped exosomes. The 120,000×*g* pellets from saliva were used for exosomes analysis. (*A*) Electron micrographs of saliva exosomes were fixed in 2% formaldehyde and contrasted using 2% uranyl acetate. The image shows small vesicles of ∼60 nm in diameter. (*B*) Exosomes were labeled with immunogold anti-CD63. Note the immunoreactivity of CD63 on the surface of the single exosome. (*C*) Representative FACS analysis of exosomes showing expression of CD63. Open trace shows the saliva exosomes, filled trace shows saliva exosomes incubated with latex beads and stained with anti-CD63 followed by secondary Alexa Fluor 488-conjugated antibody. (*D*) Western blot analysis of saliva exosomes using an antibody against CD63 and Alix. Lane 1 is the protein extract of normal saliva, and lane 2 contains protein from the exosome pellet obtained from ultracentrifugation.

Exosomes were isolated from human saliva through a series of ultracentrifugation steps with a modified version of a previously described technique [34]. Exosomes obtained from the ultracentrifuge pellets were examined by EM or stained using negative staining procedures with uranyl acetate. Electron micrographs revealed that saliva exosomes were cup-shaped, rounded vesicles of ∼30–70 nm (Fig. 1*A*) similar to exosomes released in other body fluids [13]. Notably, no visual evidence of contamination with other membrane particles was observed in our exosome preparations. To confirm the structures studied were exosome-specific, the exosomes were labeled with immunogold antibody and examined by EM (Fig. 1*B*). Antibody to the tetraspanin molecule CD63 was used, as this molecule is a commonly used marker for exosomes. The exosomes isolated from saliva appeared as electron-dense membranous structures with an average diameter of 30–100 nm and abundant CD63 immunoreactivity on the surface (Fig. 1*B*). Furthermore, FACS analysis showed enrichment of CD63 (trace peak) (Fig. 1*C*). Finally, Western blot analysis of saliva exosomes confirmed the presence of CD63 and Alix proteins in the ultracentrifugation pellets (Fig. 1*D*) and revealed that saliva-derived vesicles are positive for CD63 and Alix, confirming that the vesicles are indeed exosomes.

### AFM Analysis of Saliva Exosomes

**Figure 2:**
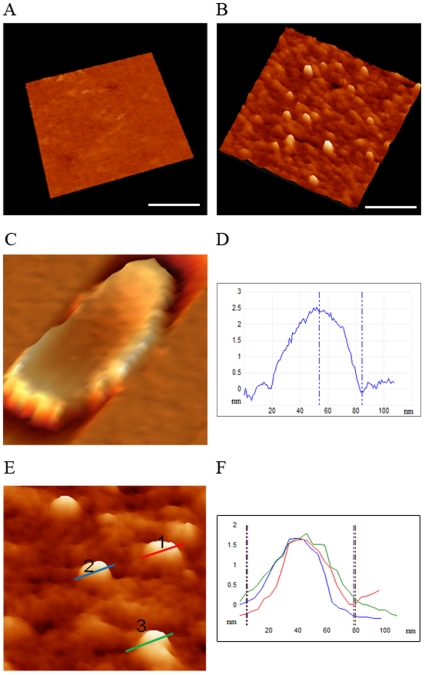
AFM images of saliva exosomes. Exosomes (panels *B*–*F*) were adsorbed to WGA-coated mica surfaces. (*A*) Topography images were obtained with the use of the Mac mode in water (negative control—no exosomes). (*B*) A 3D AFM image of isolated exosomes adhering to a mica sheet. The bar denotes 200µM. (*C*) A high-resolution single image of the exosome structure on the mica. (*D*) Graphical representation of height and width of a single exosome. (E) Size distribution of several saliva exosomes imaged with AFM. (*F*) Graphical representation of the size distribution of exosomes showing near homogeneity with respect to height and width.

While EM is a standard technique for exosome characterization, this technique may not provide a representative view of the exosomes due to the inherently harsh sample processing requirements. To assess the native exosome structure, we used AFM to study the size and structure of individual isolated vesicles. Isolated exosomes were immobilized on a mica surface (Fig. 2A control surface with no exosomes). Exosomes appeared as isolated vesicles with characteristic flattened donut-like structures in a 3D topographic image (Fig. 2B). A high-resolution phase image (Fig. 2C) of a single exosome revealed a contrast between the outer dense walls and the inner less dense region. As the exosomes were essentially free of cytoskeletal components, this fine structure reflects an inherent organization of the vesicular membrane itself. Measurements for the same exosome as shown in the topography image revealed a width of 65 nm and a height of 2.5 nm (Fig. 2D). Since the characteristic shape and size of exosomes is distinct from any other structures seen on the surface, the height profile of three individual exosomes and the size distribution of exosomes is shown (Fig. 2E and 2F), indicating consistent morphology. Furthermore, the size distribution shows cross-contamination of the exosomes with small vesicles due to limitations of the separation methodology. Next, we have extensively used molecular recognition spectroscopy with anti-CD63 IgG functionalized AFM tips for imaged and investigated the highly specific and sensitive detection of individual exosome in saliva (unpublished observation).

### Presence of mRNAs in Saliva Exosomes

**Figure 3:**
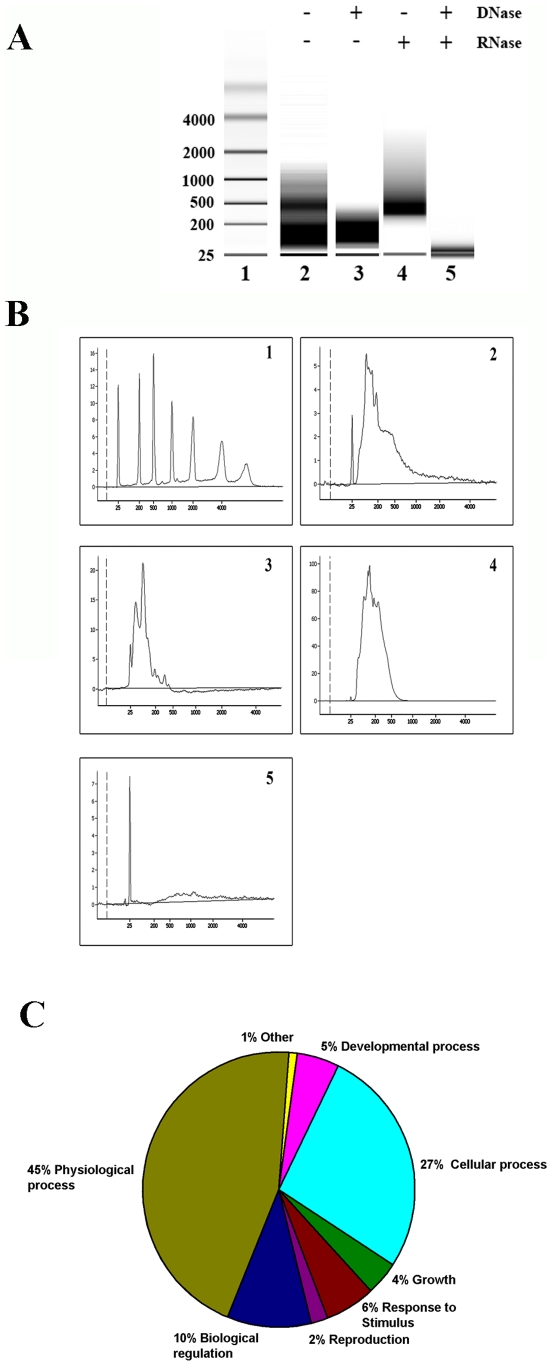
Exosomes contain mRNA species. (***A***) RNA from saliva exosomes was detected using an Agilent bioanalyser. Lane 1, RNA ladder showing sizes of the nucleotides on the left. Representative lanes (2–5) showing sizes of the mRNA species identified using an Agilent bioanalyser electrophorogram. The saliva exosomal RNA contains no ribosomal RNA as seen by the small heterogeneous RNA fragments (<200 nucleotides). (***B***) Bioanalyzer graphical data shows the size distribution of total RNA extracted from saliva exosomes (**1**) profile of RNA standard (**2**) total RNA extracted from saliva exosomes without any treatments (**3**) total RNA treated with DNase (**4**) total RNA treated with RNase A and (**5**) total RNA treated with both DNase and RNase A. (***C***) The biological process ontology of the 509 core mRNA species identified in the saliva exosomes.

The presence of nucleic acids in saliva was examined to determine a potential mechanism by which exosomes exchange genetic information. Assessment of isolated exosomes showed that these vesicles contained a substantial amount of RNA and not much DNA (Fig. 3A). Treatment with RNase A and DNase revealed the presence of mRNA in the saliva exosome preparation (Fig. 3A lanes 3 and 4). The extracted total RNA did not contain intact ribosomal RNA, as most of the ribosomal RNA was degraded, heterogeneous in nature, and <200 nucleotides in length (Fig. 3B 1–5). These observations are consistent with previous data which indicate no or minimal ribosomal RNA in exosomes [29]. Microarray assessment of saliva exosomes revealed 509 core mRNA transcripts (see supplementary data Table S1) that were common to four different microarray chip data sets (each set was derived from pooled whole saliva samples from six healthy subjects). A gene ontology-based analysis (www.bioinfo.vanderbilt.edu/gotm) implicated the saliva exosome RNA in various biological processes, including cellular and physiological processes (Fig. 3C).

### Exosome mRNA Is Stable

**Figure 4:**
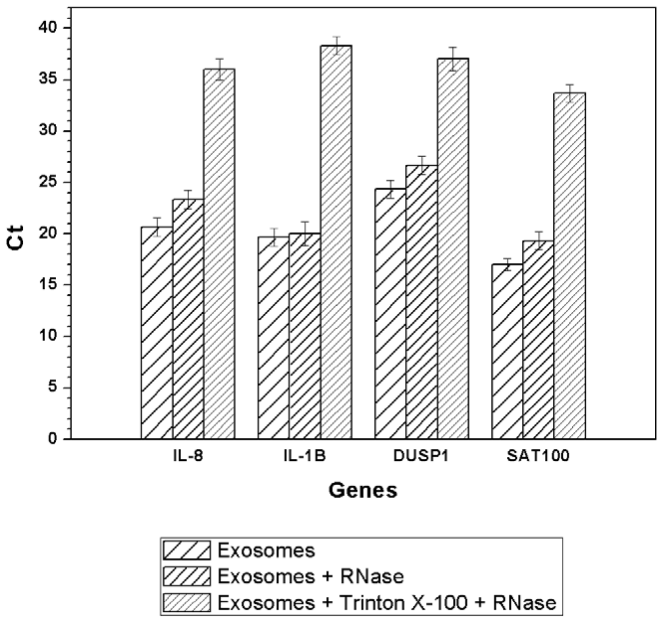
Saliva exosomes treated with Triton X-100 and RNase had different RNA content compared to control, indicating that RNA is protected inside the exosomes. Higher Ct values represent lower RNA content. Error bars denote SEM (n = 3).

Enrichment of sphingomyelin, GM3, and cholesterol is a characteristic of the so-called lipid raft domains [35], which are otherwise known as exosomes. Such domains are usually sensitive to solubilization with ionic detergents [36]. To determine whether saliva exosomes display lipid raft-like properties, we determined their solubility in the presence of 1% Triton X-100 followed by extraction and quantification of RNA by qRT-PCR (Fig. 4). The solubility of the exosomes was greater in Triton X-100 than without detergent or in NP-40 (data not shown). To confirm that the RNA is confined within the exosomes, detergent (Triton X-100) and RNase treatment of the saliva exosomes was performed at 37°C, and then the RNA was extracted as described in §Materials and Methods. No differences in RNA decay were observed between the RNase-treated and control exosomes, whereas treatment with Triton X-100 followed by RNase treatment destroyed the RNA molecules (Fig. 4). These observations are consistent with our previous observations on the stability of mRNA in whole saliva [23].

### Saliva Exosomes Are Biologically Functional

**Figure 5:**
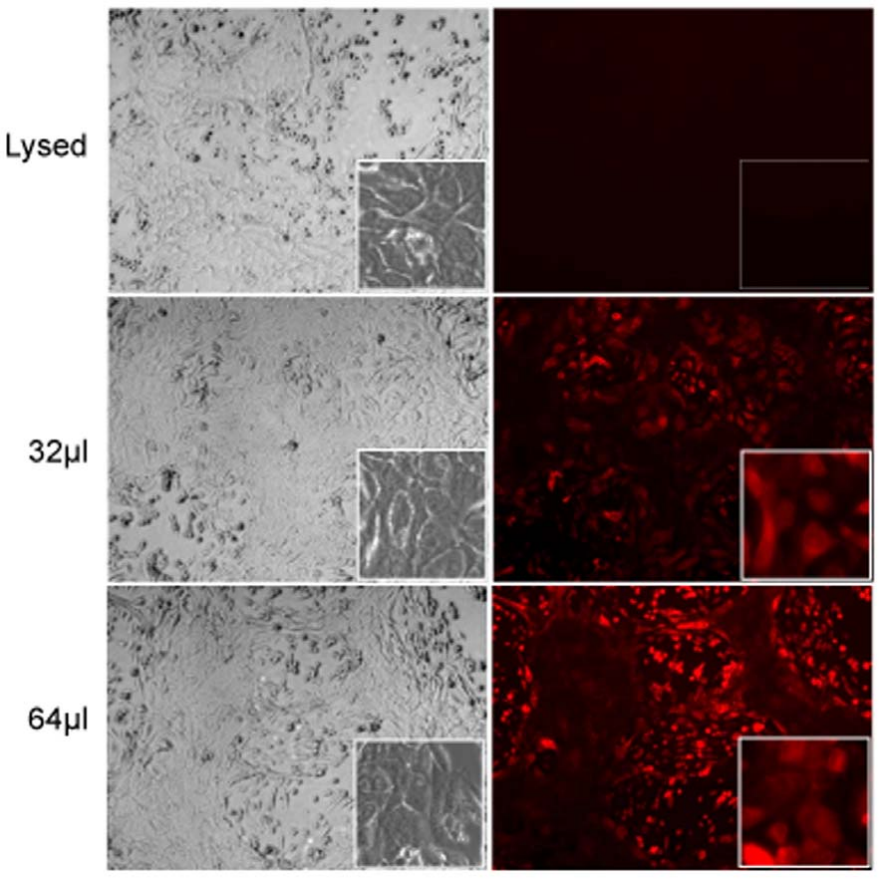
Oral keratinocytes (5×10^7^ cells/well) were incubated for 24 hr in KSFM media with fluorescently labeled exosomes and examined under fluorescence microscopy. The lysed lanes serve as a negative control. Magnification was 10×, and the smaller boxed panels represent magnification of 40×. Note the fluorescence intensity increases with increasing amounts of exosomes (32 and 64 µl, respectively).

The transfer of mRNA from saliva exosomes to human oral keratinocytes was demonstrated using vesicles derived from saliva that was labeled with fluorescent lipid BODIPY-PC. The labeled exosomes were incubated with keratinocytes and BODIPY-PC-positive exosomes transferred to recipient cells sufficiently for detection by fluorescence microscopy (Fig. 5, panels 1–4). The increasing concentration of labeled exosomes incorporated into oral keratinocytes confirmed the cell-cell communication between these two partners. The negative control detergent lyses of exosomes did not exhibit this interaction with oral keratinocytes.

**Figure 6:**
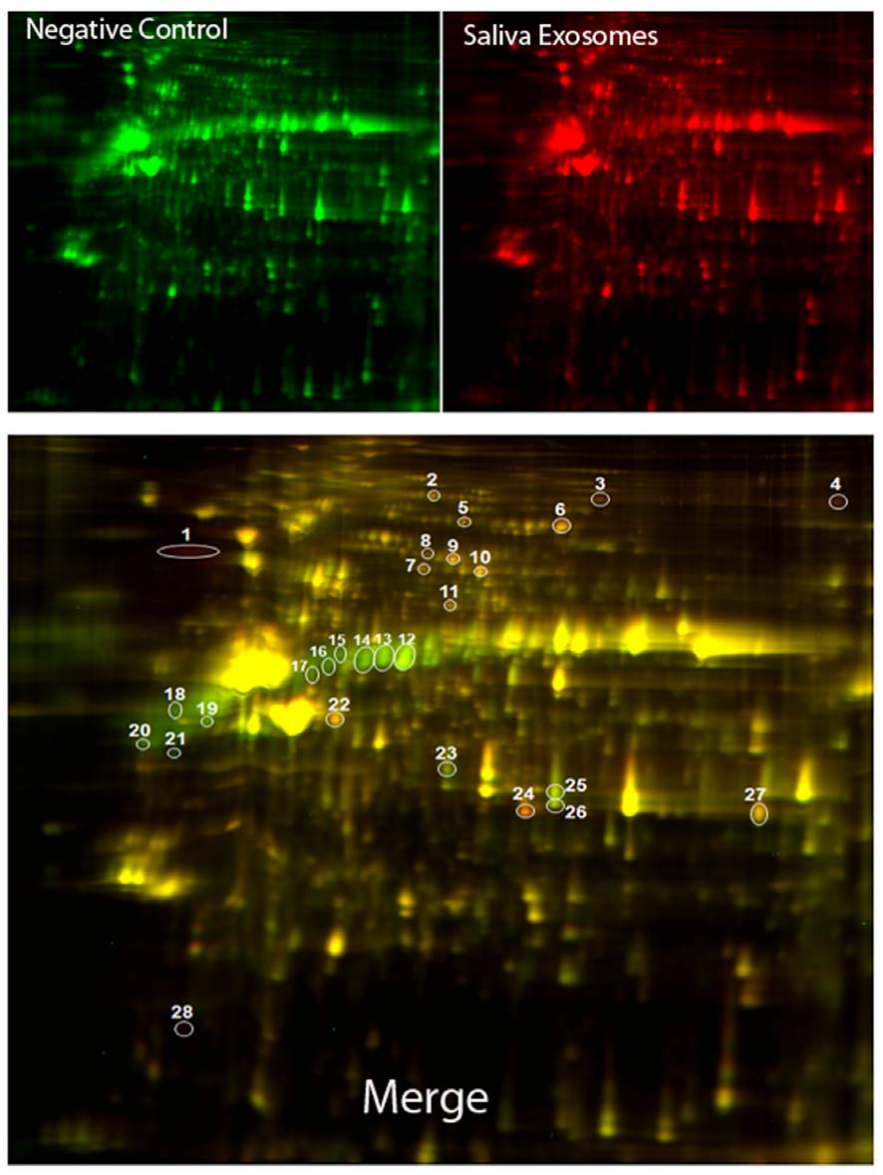
Identification of differentially expressed proteins from oral keratinocytes treated with saliva exosomes by 2-DIGE. Proteins from cells treated with the negative control were labeled with Cy3 (*green*) and proteins from cells treated with saliva exosomes were labeled with Cy5 (*red*). Isoelectric focusing was carried out at pH 3–10, and 2D separation was performed with 8–14% gradient SDS-PAGE. The negative control represents protein profiles of keratinocytes treated with detergent lysed exosomes. The bottom gel image reveals differentially expressed proteins in the control and treated samples after merging. Protein spots shown in *red* are presumably due to upregulation by exosome treatment, and those in *green* are due to downregulation by exosome treatment. Such spots are *circled* and *numbered*.

**Figure 7:**
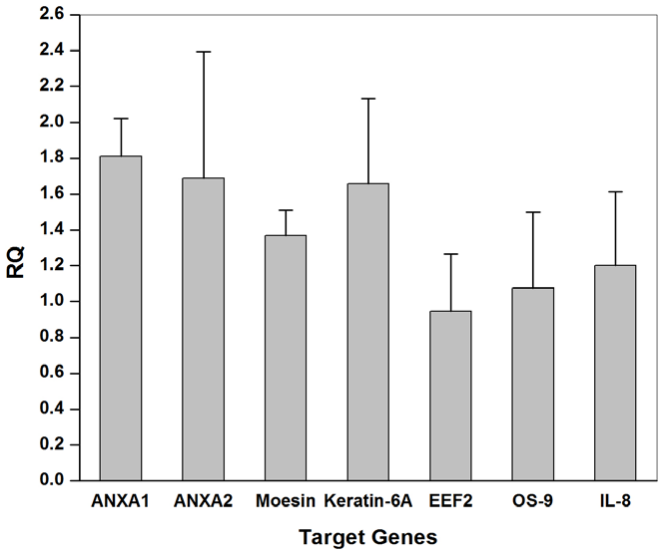
Protein expression modulated in saliva exosomes. ANXA1, ANXA2, moesin, keratin-6A, EEF2, OS-9, and IL-8 RNA expression was normalized using a factor calculated from β-Actin gene expression.

Although exosomes obtained from saliva of healthy individuals suggested a role in the oral microenvironment, the underlying mechanism of salivary exosomes interaction is still unclear largely because of limited information about saliva exosomes. Surprisingly, several reports have predicted cell-cell communication functions of exosomes, but saliva exosomes have not been examined experimentally. Because exosomes are thought to regulate gene expression in recipient cells, we determined the differential expression of proteins in keratinocytes after treatment with saliva exosomes. Protein was extracted from keratinocytes treated with either a negative control of detergent treated exosomes and labeled with Cy3 or saliva exosomes labeled with Cy5. Unlike conventional two-dimensional gels in which the two samples are run in separate gels, we separated the two samples, which were labeled with different fluorescent dyes, in a single gel, thus eliminating gel-to-gel variation and allowing for easy comparison of relative expression levels. After separation, several proteins were either upregulated or downregulated (Fig. 6, red or green, respectively). This result, in fact, is in agreement with data indicating that mast cell-derived exosomes also up- or down-regulate many proteins [29]. We are particularly interested in those proteins that are differentially regulated by saliva exosomes, because those are potentially direct targets for exosomes. Nine protein spots with more than 2-fold expression changes in keratinocytes treated with exosomes compared with the negative control were chosen for closer examination (Fig. 6, indicated by circles and numbers). Mass spectrometry analysis identified all nine of these proteins with a good score (Table 1). Next we investigated whether the modulated gene transcripts are present in the saliva exosomes and could possibly be involved in the translation. To identify these specific genes in exosomes, we analyzed RNAs extracted from the respective saliva exosomes using quantitative RT–PCR. The housekeeping gene β-actin was used a reference control, and the relative quantity of mRNA targets were measured in saliva exosomes (Fig. 7). As noted by others, genes for annexin A1, annexin A2, moesin, keratin-6A, eukaryotic elongation factor-2, OS-9 and interleukin-8 are present in exosomes, suggesting that possible translation of proteins occurs in the recipient keratinocytes [29]. In other words, altered protein expression by recipient cells could be an ongoing translation of these mRNAs. These data suggest that the RNA incorporated into the exosomes may be delivered into the recipient cells and generate a functional protein.

**Table 1:** Proteins expressed differentially in oral keratinocytes.

Spot number	Protein name	Accession No.	Protein MW	Protein Abundance	Peptide count	Functional Annonation
1	OS-9	gi|48145699	69189.4	Increased	9	*Protein binding*
6	Eukaryotic translation elongation factor 2	gi|4503483	95277	Increased	29	*GTP binding, translation elongation activity*
12	Keratin 6A	gi|15559584	59981.3	Decreased	8	*Structural molecule activity*
22	Serine (or cysteine) proteinase inhibitor, clade B (ovalbumin), member 2	gi|4505595	46566.1	Increased	22	*Serine-type endopeptidase activity*
24	Annexin A1	gi|54696696	38690	Increased	16	*Calcium/phospholipid-binding protein, Involved in exocytosis*
25	Annexin A2	gi|30583703	38579.8	Decreased	18	*Calcium binding protein, involved in exocytosis*
27	Human Muscle L-Lactate Dehydrogenase M Chain	gi|13786856	36534.3	Increased	17	*dehydrogenase activity, protein binding*
10	Moesin	gi|5419633	67777.8	Increased	31	*Cytoskeletal protein binding*

Thus, here we show that exosomes are formed similarly to other previously identified types of MVBs. Microarray analysis revealed the presence of 509 mRNA transcripts known to be present in exosomes, confirming the observation that exosomes in saliva contain RNA. In addition, experimental *in vitro* transfer of saliva exosomes altered the gene expression of recipient oral keratinocytes. Together, these studies demonstrate that saliva exosomes are biologically active and may potentially be a useful agent in studies aimed at disease diagnostics and therapeutics.

## Discussion

Exosomes and their genetic contents can regulate a variety of cellular pathways through regulation of the expression of multiple target genes in recipient cells [31]. In this regard, exosomes have been suggested to function as immune-response modifiers because these vesicles are secreted by many types of tumors cells. Exosomes were previously found to be secreted in saliva [12]; although, no physiological function was assigned. Exosomes are released into the saliva via either ductal or acinar cells [37]. Essentially salivary glands have been implicated in a constitutive-like secretory pathway involved in secretion of exosomal-like vesicles. These secretory vesicles are derived directly from the trans-Golgi or involve elements of the endosomal-lysosomal trafficking pathway [38]. In this study, we isolated saliva exosomes and showed that these vesicles were, in fact, physiologically active. Consistent with previous EM images of exosomes in body fluids [13], [16], [18], ultrastructural examination of saliva exosomes revealed small vesicles with diameters <100 nm and a unique “cup-like” shape, which are both characteristic features of exosomes. AFM also revealed the ultrastructural features and distribution of the exosomes.

In addition, microarray analysis indicated the presence of mRNA inside the exosomes, and these nucleic acids were protected against ribonucleases in saliva. Furthermore, the exosomal RNA analysis of Valadi *et al.* [29] demonstrated that virtually no ribosomal RNA was present and that most of the RNA molecules were <200 nucleotides in length. Moreover, saliva exosome RNA exhibited characteristic features similar to mast cell-derived exosomal RNA. Finally, RNA present in exosomes was functional as modulation of gene expression was observed in keratinocytes incubated with the exosomes. This finding was in accord with recent reports that exosomes can transfer mRNA horizontally to neighboring cells [24], [39]. The notion that exosome RNA is delivered to other cells provides added functional significance to salivary exosomes.

The functions of exosomes should be reflected by their proteins and mRNA molecules, which originate from endocytic release. Because exosomes are formed as MVBs, these particles likely contain factors required for MVB formation and protein sorting. Analysis of exosomes derived from human mast cells, dendritic cells, and epithelial cells as well as other cell types revealed the presence of common and cell type-specific proteins and mRNA. For example, the aquaporin family of proteins was especially enriched in exosomes derived from body fluids such as urine and amniotic fluid [13], [14]. Human saliva and saliva exosomal proteins have been identified and cataloged in detail [11], [40], [41] including aquaporins, cytoskeleton proteins, and membrane proteins, which have also been found in exosomes from other cell types. Importantly, sorting of disease-specific proteins into exosomes is quite useful for diagnostic applications [9]. The molecular factors and mechanisms behind this cell-specific sorting process in exosomes are still unknown, and such an analysis may help their translational utility.

Proteomics analysis of saliva ductal fluids revealed 1,166 proteins, including various membrane-bound proteins [40]. Interestingly, the aquaporin protein family, which is involved in water flow through membranes, was identified in saliva. Aquaporins have been identified on both the apical and basolateral membranes of secretory acinar cells of salivary glands [37]. Surprisingly, AQP1 and AQP2 proteins identified in urine exosomes via secretion through renal ductal cells were implicated in pathophysiological processes in urinary epithelial cells [13]. Additionally, decreases in aquaporin expression are linked to various kidney and pancreas diseases, while reduced aquaporin expression in salivary glands is linked to Sjögren's syndrome [42]. Furthermore, the annexin family of proteins bind to intracellular membranes and is involved in intracellular membrane fusion [43]. Association of annexins with exosomes may result from the presence of phosphatidylserine in these vesicles [44]. Interestingly, Annexins and Alix proteins are reportedly present in saliva exosomes [11]. Differential expression of annexin A1, annexin A2, moesin, and OS-9 proteins indicated the influence of saliva exosomes in oral keratinocytes. Interestingly, presence of annexin A1 mRNA in saliva exosomes may translate protein in the recipient cell gene expression. In addition, moesin, which is an actin-binding protein of the ERM family in exosomes, has been demonstrated to play a role in *de novo* actin assembly on phagosomal membranes [45]. Further, moesin has been reported to be present in B cell-derived exosomes [46] and breast milk [18].

Clearly, exosome-like microvesicles are present in body fluids such as saliva, blood, amniotic fluid, and pleural effusions under both healthy and disease conditions; however, the origin of these exosomes and their intended destination for stimulation of distal cells remains unclear. Here, we demonstrated that saliva exosomes can be taken up by oral keratinocytes. Interestingly, our observation establishes another dimension of cell-cell communication of body fluid exosomes. Notably, keratinocytes are able to secrete exosomes and externalize stratifin protein, which is a potent stimulant of metalloproteinases in fibroblasts [47]. Arguably, both keratinocytes and saliva exosomes engage in cell-cell communication, and the possibility exists that part of the saliva exosomes originates from oral keratinocytes. Whether these interactions are involved in a novel mechanism of cell-cell communication is an intriguing, yet unanswered, question. Our studies do not directly identify the functional consequence of mRNA release via exosomes; however, saliva exosomes carrying mRNA transcripts of these specific altered proteins suggest that these RNAs could possibly be translated into proteins at their new location. Also, recent studies suggest an important role for exosomes in the modulation of host gene expression levels. Interestingly, exosomes purified from mast cells [29] and neuronal cells [24] are enriched in mRNA molecules that stimulate and alter gene expression of recipient cells. These data have led to the suggestion that secreted exosomes expressing relevant mRNAs may play a role in the generation of new genes and modulate gene expression of recipient cells. Indeed, annexin and moesin are overexpressed in keratinocytes following incubation with saliva exosomes. We cannot rule out the presence of lipids and proteins in saliva exosomes that also can trigger gene expression at their new recipient cells. We have observed several ceramide lipid species in saliva exosomes that could potentially have impact on oral keratinocytes (unpublished observation). Finally, the source of these exosomes in saliva, however, is probably heterogeneous, and formal demonstration that salivary glands secrete exosomes *in vivo* awaits further analyses.

In summary, saliva exosomes may regulate cell-cell environment by altering their gene expression. This study extends our knowledge about human saliva exosomes. In addition to genetic regulation, as mentioned above, saliva exosomes are involved in protecting nucleic acids against nucleases in the oral cavity. Thus, saliva exosomes, like other types of exosomes, clearly have multiple functions. We expect that more saliva exosome targets will be identified in the near future using the same proteomic approach for various systemic diseases. These discoveries will allow us to better understand the molecular basis of oral diseases. The studies of Valadi [29], Skog [30], and Ratajczak [39] as well as the present study open up a new research perspective on the use of exosomal transfer of mRNA to target another cell type. In particular, the results of the present study indicate that exosomes derived from human saliva activate or modulate gene expression in oral keratinocytes.

## Methods

In this study, we employed several molecular approaches including EM and AFM to characterize saliva exosomes. Next, we determined whether RNAs present in saliva exosomes are protected and whether this genetic information is shared between cells.

### Participants

Saliva samples were obtained from healthy volunteers from the Division of Otolaryngology, Head, and Neck Surgery, at the Medical Center, University of California, Los Angeles (UCLA), CA in accordance with a protocol approved by the UCLA Institutional Review Board. All participants gave written informed consent, and the ethics committee of UCLA approved the study. The mean age of the volunteers was 31 years (range 26–43 years). The volunteers had no history of malignancy, immune deficiencies, autoimmune disorders, hepatitis, or HIV infection.

### Purification of Exosomes

Exosomes were prepared as described [34] with slight modifications. Briefly, 50 ml of saliva was mixed with an equal volume of PBS and centrifuged at 2600×*g* for 15 min to remove cells. The supernatants were then sequentially centrifuged at 12,000×*g* for 20 min and 120,000×*g* for 3 hours. The final pellet was resuspended in PBS and then used either for immunoblotting or for EM. Notably, saliva is highly viscous in nature and it is very difficult to apply filtration procedures with specific membrane filters (0.2 µM or 0.45 µM size) before it undergoes ultracentrifugation.

### Immunoelectron Microscopy

The isolated exosomes were loaded onto carbon-coated grids, fixed in 2% paraformaldehyde, washed, and then immunolabeled with anti-CD63 antibody (Santa Cruz Biotechnology, Santa Cruz, CA) followed by a 10 nm gold-labeled secondary antibody (Sigma Aldrich, St. Louis, MO). The exosomes were post-fixed in 2.5% glutaraldehyde, washed three times, contrasted with 2% uranyl acetate, and then examined with a JEOL 100CX transmission electron microscope (JEOL USA, Inc. Peabody, MA).

### Atomic Force Microscopy

For AFM imaging of isolated exosomes purified samples were diluted 1∶100 in deionized water and adsorbed to freshly cleaved mica sheets for 10 min. The sheets were rinsed thoroughly with deionized water to remove unbound exosomes and dried under a gentle stream of nitrogen. Bioscope II (Veeco Digital Instruments, Santa Barbara, CA) was used for tapping mode AFM imaging using silicon probes with spring constant *k* = 305 KHz (OTESPA, Veeco). Topographic height and phase images were recorded simultaneously at 512×512 pixels at a scan rate of 0.4 Hz. The height of the exosomes was obtained from a line profile of height images (Nanoscope software). Image processing was performed using the WxSM free software (Nanotec, Spain).

### Immunoblotting and Flow Cytometry

Exosomal proteins were extracted and separated on a polyacrylamide gel before transfer to a nitrocellulose membrane. The blotting membrane was blocked and incubated with CD63 antibody followed by incubation with horseradish peroxidase-coupled secondary antibody. The proteins were detected using enhanced chemiluminescence. For FACS analysis, saliva exosomes were absorbed onto 4-µm aldehyde-sulfate latex beads (Interfacial Dynamics, OR) and incubated with CD63 antibody and/or Alix (Santa Cruz Biotechnology, Santa Cruz, CA) followed by an incubation with a secondary antibody (Molecular Probes, Invitrogen, CA). The exosomes were then washed and analyzed on a FACSscan (BD Biosciences, San Diego, CA).

### RNA Isolation and Amplification

RNA was isolated from 300 µg of saliva exosomes with the RNeasy Mini Kit (Qiagen, Valencia, CA), according to the manufacturer's instructions. All samples were treated with TURBO DNA-*free* (Ambion, Austin, TX) to remove trace amounts of genomic DNA. A 2-round amplification was performed with the RiboAmp RNA Amplification Kit (Molecular Devices, Sunnyvale, CA) according to the manufacturer's instructions.

### RNA Detection and Analysis

Detection of RNA was achieved using the Agilent 2100 Bioanalyser (http://www.chem.agilent.com). Triton X-100 (Sigma) was added at a final concentration of 1%, and exosomes were incubated at room temperature for up to 30 min with and without RNase A and DNase at a final concentration of 100 units/ml. After RNA isolation, additional water and Triton X-100 were added to the respective samples to balance the chemical composition.

### Microarray Analysis

The microarray experiments were performed by the UCLA microarray core facility according to the Affimetrix microarray analysis protocols. Briefly, single-stranded cDNA was generated from the amplified cRNA with the WT cDNA Synthesis Kit (Affymetrix, Santa Clara, CA) and then fragmented and labeled with the WT Terminal Labeling Kit (Affymetrix). Samples were hybridized with GeneChip HGU-133 plus 2 Arrays (Affymetrix) and scanned at the UCLA Microarray Core Facility. Raw data were processed with the Quantile normalization (part of GCRMA package). A detection *p*-value was obtained for each probe set. Any probe set with *p*<0.04 was assigned as “present”, indicating that the matching gene transcript was reliably detected. The total number of present probe sets on each array was obtained, and the percentage (P%) of present genes was calculated. The program R-package was used for gene profiling analysis, and the gene ontology software was used for the biological process analysis.

### RT-PCR Preamplification

Multiplex RTPCR preamplifications were performed in 10-µL reaction volumes with a pool of outer primers at 300 nmol/L each and the SuperScript III Platinum One-Step qRT-PCR System (Invitrogen). Reactions were prepared on ice, loaded into a preheated thermocycler, and performed as follows: 1 min at 60°C, 15 min at 50°C, 2 min at 95°C, and 15 cycles of 15 s at 95°C, 30 s at 50°C, 10 s at 60°C, and 10 s at 72°C. These steps were followed with a final extension of 5 min at 72°C and cooling to 4°C. Immediately after the RT-PCR, we treated 5 µL of the reaction with 2 µL of ExoSAP-IT (USB Corporation) for 15 min at 37°C to remove excess primers and deoxynucleoside triphosphates and then heated the mixtures to 80°C for 15 min to inactivate the enzyme mix. The preamplification products were then diluted 40-fold with water to 200 µL to enable qPCR analysis of all targets.

### Quantitative PCR

Each transcript was quantified from 2 µL aliquots of preamplified samples via a singleplex qPCR in an SDS 7500 Fast instrument (Applied Biosystems, Foster City, CA) with a 10-µL reaction volume containing 300 nmol/L of each of the inner primers and the SYBR Green Power Master Mix (Applied Biosystems). After 10 min of polymerase activation at 95°C, we carried out 40 cycles of 15 s at 95°C and 60 s at 60°C and then performed a melting curve analysis. Table S2 shows the primers sets used for this study (supplementary methods).

### *In Vitro* Labeling of Exosomes

The *in vitro* labeling of exosomes was performed as described [48] with slight modification. Briefly, purified saliva exosomes were incubated with 10 µM of BODIPY-PC in PBS for 30 min at 37°C in the dark. Excess fluorescent lipids were removed by ultracentrifugation at 120,000×*g* for 1.5 hours at 4°C. Labeled exosomes were then resuspended with PBS and then used for *in vitro* transfer experiments.

### Cells and *In Vitro* Transfer Experiments

Immortalized human oral keratinocytes (OKF6tert1) were cultured and harvested under log phase conditions as described previously [49]. For *in vitro* transfer experiments, the labeled saliva exosomes were added to oral keratinocytes (2×10^7^) at a final concentration of 2 mg/ml. At 0 hr and 24 hr, cells were harvested and washed three times. Total cellular proteins were extracted and separated by 2D electrophoresis. A sample with 1% Triton X-100 of lysed exosomes was treated similarly and used as a negative control.

### Accession Numbers

Details about the microarray data deposition can be found at http://www.ncbi.nlm.nih.gov/geo (the GEO accession number is: GSE13494). All the data obtained from Microarray are in accordance with MIAME compliant, as detailed on the website www.mged.org/workgroups/MIAME.miame.html.

### Proteomic Analysis of Exosomes Transfer Experiment Samples

Oral keratinocytes that were harvested after incubation with exosomes were sent directly for 2-DIGE and mass spectrometry analysis by Applied Biomics (Hayward, CA) Briefly, total protein was extracted, labeled with Cy3 and Cy5 dyes (GE Healthcare, Piscataway, NJ), and subjected to isoelectric focusing (pH 3–10) and sodium dodecyl sulfate–polyacrylamide gel electrophoresis (SDS-PAGE). Gel scanning was carried out immediately after SDS-PAGE using Typhoon TRIO (GE Healthcare, Piscataway, NJ). Scanned images were analyzed by Image Quant software (version 5.0, GE Healthcare) and subjected to in-gel analysis and cross-gel analysis using the DeCyder software (version 6.0, GE Healthcare) with a detection limit of 0.2 ng of protein per spot. The ratio change for differentially expressed protein spots was obtained from the in-gel DeCyder analysis. Protein spots of interest that were consistently differentially expressed in exosomes- versus vehicle-treated samples across a minimum of two SDS gels were picked up by Ettan Spot Picker (GE Healthcare, Piscataway, NJ) and subjected to in-gel trypsin digestion, peptide extraction, and desalting prior to MALDI-TOF/MS-MS (ABI 4700, Applied Biosystems, CA). Peptide fingerprints and partial amino acid sequence information were used for protein identification in the nrNCBI nonredundant National Center for Biotechnology) databases. Searches were performed without constraining protein molecular weight (MW) or isoelectric point (IP), with variable carbamidomethylation of cysteine and oxidation of methionine residues, and with one missed cleavage allowed in the search parameters. Candidates with protein and ion scores greater than 95% were considered significant.

### Statistical Analysis

Data are expressed as means±SEM and *P* values <0.01 were considered statistically significant according to the Student's *t* test.

## Supporting Information

Table S1 Supplementary data
(0.08 MB XLS)

Table S2 Supplementary methods
(0.03 MB DOC)

## References

[1] Delcayre A, Estelles A, Sperinde J, Roulon T, Paz P (2005). Exosome Display technology: applications to the development of new diagnostics and therapeutics.. Blood Cells Mol Dis.

[2] Garcia JM, Garcia V, Pena C, Dominguez G, Silva J (2008). Extracellular plasma RNA from colon cancer patients is confined in a vesicle-like structure and is mRNA-enriched.. RNA.

[3] Heijnen HF, Schiel AE, Fijnheer R, Geuze HJ, Sixma JJ (1999). Activated platelets release two types of membrane vesicles: microvesicles by surface shedding and exosomes derived from exocytosis of multivesicular bodies and alpha-granules.. Blood.

[4] Rozmyslowicz T, Majka M, Kijowski J, Murphy SL, Conover DO (2003). Platelet- and megakaryocyte-derived microparticles transfer CXCR4 receptor to CXCR4-null cells and make them susceptible to infection by X4-HIV.. AIDS.

[5] Blanchard N, Lankar D, Faure F, Regnault A, Dumont C (2002). TCR activation of human T cells induces the production of exosomes bearing the TCR/CD3/zeta complex.. J Immunol.

[6] Lamparski HG, Metha-Damani A, Yao JY, Patel S, Hsu DH (2002). Production and characterization of clinical grade exosomes derived from dendritic cells.. J Immunol Methods.

[7] Bhatnagar S, Shinagawa K, Castellino FJ, Schorey JS (2007). Exosomes released from macrophages infected with intracellular pathogens stimulate a proinflammatory response in vitro and in vivo.. Blood.

[8] Escola JM, Kleijmeer MJ, Stoorvogel W, Griffith JM, Yoshie O (1998). Selective enrichment of tetraspan proteins on the internal vesicles of multivesicular endosomes and on exosomes secreted by human B-lymphocytes.. J Biol Chem.

[9] Kapsogeorgou EK, Abu-Helu RF, Moutsopoulos HM, Manoussakis MN (2005). Salivary gland epithelial cell exosomes: A source of autoantigenic ribonucleoproteins.. Arthritis Rheum.

[10] Iero M, Valenti R, Huber V, Filipazzi P, Parmiani G (2008). Tumour-released exosomes and their implications in cancer immunity.. Cell Death Differ.

[11] Gonzalez-Begne M, Lu B, Han X, Hagen FK, Hand AR (2009). Proteomic Analysis of Human Parotid Gland Exosomes by Multidimensional Protein Identification Technology (MudPIT).. J Proteome Res.

[12] Ogawa Y, Kanai-Azuma M, Akimoto Y, Kawakami H, Yanoshita R (2008). Exosome-like vesicles with dipeptidyl peptidase IV in human saliva.. Biol Pharm Bull.

[13] Pisitkun T, Shen RF, Knepper MA (2004). Identification and proteomic profiling of exosomes in human urine.. Proc Natl Acad Sci U S A.

[14] Keller S, Rupp C, Stoeck A, Runz S, Fogel M (2007). CD24 is a marker of exosomes secreted into urine and amniotic fluid.. Kidney Int.

[15] Runz S, Keller S, Rupp C, Stoeck A, Issa Y (2007). Malignant ascites-derived exosomes of ovarian carcinoma patients contain CD24 and EpCAM.. Gynecol Oncol.

[16] Prado N, Marazuela EG, Segura E, Fernandez-Garcia H, Villalba M (2008). Exosomes from bronchoalveolar fluid of tolerized mice prevent allergic reaction.. J Immunol.

[17] Simpson RJ, Jensen SS, Lim JW (2008). Proteomic profiling of exosomes: current perspectives.. Proteomics.

[18] Admyre C, Johansson SM, Qazi KR, Filen JJ, Lahesmaa R (2007). Exosomes with immune modulatory features are present in human breast milk.. J Immunol.

[19] Raab A, Han W, Badt D, Smith-Gill SJ, Lindsay SM (1999). Antibody recognition imaging by force microscopy.. Nat Biotechnol.

[20] Li Y, St John MA, Zhou X, Kim Y, Sinha U (2004). Salivary transcriptome diagnostics for oral cancer detection.. Clin Cancer Res.

[21] Li Y, Zhou X, St John MA, Wong DT (2004). RNA profiling of cell-free saliva using microarray technology.. J Dent Res.

[22] Palanisamy V, Park NJ, Wang J, Wong DT (2008). AUF1 and HuR proteins stabilize interleukin-8 mRNA in human saliva.. J Dent Res.

[23] Park NJ, Li Y, Yu T, Brinkman BM, Wong DT (2006). Characterization of RNA in saliva.. Clin Chem.

[24] Deregibus MC, Cantaluppi V, Calogero R, Lo Iacono M, Tetta C (2007). Endothelial progenitor cell derived microvesicles activate an angiogenic program in endothelial cells by a horizontal transfer of mRNA.. Blood.

[25] Lakkaraju A, Rodriguez-Boulan E (2008). Itinerant exosomes: emerging roles in cell and tissue polarity.. Trends Cell Biol.

[26] Schorey JS, Bhatnagar S (2008). Exosome function: from tumor immunology to pathogen biology.. Traffic.

[27] Fackler OT, Peterlin BM (2000). Endocytic entry of HIV-1.. Curr Biol.

[28] Fevrier B, Raposo G (2004). Exosomes: endosomal-derived vesicles shipping extracellular messages.. Curr Opin Cell Biol.

[29] Valadi H, Ekstrom K, Bossios A, Sjostrand M, Lee JJ (2007). Exosome-mediated transfer of mRNAs and microRNAs is a novel mechanism of genetic exchange between cells.. Nat Cell Biol.

[30] Skog J, Wurdinger T, van Rijn S, Meijer DH, Gainche L (2008). Glioblastoma microvesicles transport RNA and proteins that promote tumour growth and provide diagnostic biomarkers.. Nat Cell Biol.

[31] Smalheiser NR (2007). Exosomal transfer of proteins and RNAs at synapses in the nervous system.. Biol Direct.

[32] Denzer K, Kleijmeer MJ, Heijnen HF, Stoorvogel W, Geuze HJ (2000). Exosome: from internal vesicle of the multivesicular body to intercellular signaling device.. J Cell Sci.

[33] Thery C, Zitvogel L, Amigorena S (2002). Exosomes: composition, biogenesis and function.. Nat Rev Immunol.

[34] Thery C, Amigorena S, Raposo G, Clayton A (2006). Isolation and characterization of exosomes from cell culture supernatants and biological fluids.. Curr Protoc Cell Biol Chapter.

[35] Ikonen E (2001). Roles of lipid rafts in membrane transport.. Curr Opin Cell Biol.

[36] Brown DA, London E (2000). Structure and function of sphingolipid- and cholesterol-rich membrane rafts.. J Biol Chem.

[37] McManaman JL, Reyland ME, Thrower EC (2006). Secretion and fluid transport mechanisms in the mammary gland: comparisons with the exocrine pancreas and the salivary gland.. J Mammary Gland Biol Neoplasia.

[38] Stinchcombe JC, Griffiths GM (1999). Regulated secretion from hemopoietic cells.. J Cell Biol.

[39] Ratajczak J, Miekus K, Kucia M, Zhang J, Reca R (2006). Embryonic stem cell-derived microvesicles reprogram hematopoietic progenitors: evidence for horizontal transfer of mRNA and protein delivery.. Leukemia.

[40] Hu S, Xie Y, Ramachandran P, Ogorzalek Loo RR, Li Y (2005). Large-scale identification of proteins in human salivary proteome by liquid chromatography/mass spectrometry and two-dimensional gel electrophoresis-mass spectrometry.. Proteomics.

[41] Vitorino R, Lobo MJ, Ferrer-Correira AJ, Dubin JR, Tomer KB (2004). Identification of human whole saliva protein components using proteomics.. Proteomics.

[42] Delporte C, Steinfeld S (2006). Distribution and roles of aquaporins in salivary glands.. Biochim Biophys Acta.

[43] Gruenberg J, Emans N (1993). Annexins in membrane traffic.. Trends Cell Biol.

[44] Thery C, Boussac M, Veron P, Ricciardi-Castagnoli P, Raposo G (2001). Proteomic analysis of dendritic cell-derived exosomes: a secreted subcellular compartment distinct from apoptotic vesicles.. J Immunol.

[45] Defacque H, Egeberg M, Habermann A, Diakonova M, Roy C (2000). Involvement of ezrin/moesin in de novo actin assembly on phagosomal membranes.. EMBO J.

[46] Wubbolts R, Leckie RS, Veenhuizen PT, Schwarzmann G, Mobius W (2003). Proteomic and biochemical analyses of human B cell-derived exosomes. Potential implications for their function and multivesicular body formation.. J Biol Chem.

[47] Chavez-Munoz C, Morse J, Kilani R, Ghahary A (2008). Primary human keratinocytes externalize stratifin protein via exosomes.. J Cell Biochem.

[48] Laulagnier K, Vincent-Schneider H, Hamdi S, Subra C, Lankar D (2005). Characterization of exosome subpopulations from RBL-2H3 cells using fluorescent lipids.. Blood Cells Mol Dis.

[49] Kim Y, Shintani S, Kohno Y, Zhang R, Wong DT (2004). Cyclin G2 dysregulation in human oral cancer.. Cancer Res.

