# Supplementary material for: Nanostructural and Transcriptomic Analyses of Human Saliva Derived Exosomes
Source: PLoS One. 2010 Jan 5;5(1):e8577. doi: 10.1371/journal.pone.0008577 (PMC2797607; doi:10.1371/journal.pone.0008577)
Supplement: Table S2 — Supplementary methods (0.03 MB DOC) [file pone.0008577.s002.doc]

Supplementary methods:

Table S2. Primers used for saliva exosomes RT-qPCR.

| **Gene Target** | **Forward Primer 5′- 3′** | **Reverse Primer 5′- 3′** |
| --- | --- | --- |
| ***OS-9*** | CTTTTGAGTGGAGGTGGGTAG | CAGGGAAAGGGAGAAGGAAATC |
| ***Annexin A1*** | TCGGAACGCTTTGCTTTCTCTTGC | TTCATACAAGGCCCTGGCATCTGA |
| ***Annexin A2*** | ACGGCCCAGGTTATCTTGTAGCAT | CCAATGTGTTCAACCAAGCGGGAA |
| ***Moesin*** | ACTCAACCCTCTCAGGTGACCAAA | ACAGAACAGCTGGTGTCAAGACCT |
| ***EEF2*** | ATGTGCCTTGTGGGAACATTGTGG | AACTTCATCACCCGCATGTTGTGC |
| ***Keratin 6A*** | ACAAGGTTGAACTGCAAGCCAAGG | TGTGTCTGAGATGTGGGTCTGCAT |
| ***DUSP1*** | CCTACCAGTATTATTCCCGACG | TTGTGAAGGCAGACACCTACAC |
| ***IL1β*** | GTGCTGAATGTGGACTCAATCC | ACCCTAAGGCAGGCAGTTG |
| ***IL8*** | GAGGGTTGTGGAGAAGTTTTTG | CTGGCATCTTCACTGATTCTTG |
| ***SAT100*** | CCAGTGAAGAGGGTTGGAGAC | TGGAGGTTGTCATCTACAGCAG |
| ***Β-Actin*** | GGCACCCAGCACAATGAAG | GCCGATCCACACGGAGTA |
